# The Evolution of the Modern Hospital

**Published:** 1915-03-20

**Authors:** B. Burford Rawlings


### THE EVOLUTION OF THE MODERN HOSPITAL.*

### IV.—Medical Education, Administration, and Finance.

By B. BURFORD RAWLINGS.

Not for long together is the hospital body free from acute manifestations of chronic internal disorder. The late disagreement at St. George's, involving the resignation of the President, Princess Christian, furnished ample occasion for demanding a searching examination into the administration of Metropolitan medical charity, but the incident, like others before it, was allowed to pass. Yet the vital question whether lay or medical authority is to be supreme in philanthropic institutions which are also educational must be faced frankly, sooner or later, in the interests of the community.

What was known of the St. George's dispute warrants a belief that it occupied the old field, the question at issue being whether hospitals exist primarily for the benefit of the profession or the patients. It is because opposing interests not easily reconciled cannot be dissociated that the problem takes its most formidable aspect.

If hospitals could be wholly philanthropic or wholly educational, their administration would be spared its chief difficulty, but although the discharge of dual and sometimes conflicting functions is imperatively demanded of them, their financial and general welfare depends largely upon the assurance that their claim to benevolent support is based upon their philanthropy.

The attainment of a perfect system of education is a great and laudable object of the profession, and much can be advanced in support of the plea that the importance of hospital work is better appraised in its results to humanity at large than by reckoning its performances among the patients admitted into the wards. Nobody can doubt that the man who assists medical training by his gifts exhibits intelligent generosity, and some people would see in grants from the State to the schools a proof of enlightenment. Whether the means of medical education ought to be forthcoming is not in question; what we have to ask is how far the requirements of the schools are consistent with the exigencies of a voluntary system of support for the hospitals, and whether it is to the advantage of the institutions and their patients that they should depend upon the services of staffs whose members receive no direct payment, and are encouraged by the conditions to look for remuneration to their privileges as teachers and students?

Hospitals and schools are fated to live together, but while the hospital is indispensable to the school, the school is not quite necessary to the hospital. Some hospitals exist without schools and accomplish good work. The retort is obvious, but none the less hospitals, as a vast majority of their supporters think, exist for the benefit of the patients actually treated, and not a few donors dislike intensely the suggestion that in making their gifts they are helping agencies useful to themselves or their class. They desire that their contributions shall be free offerings to the poor. A well-known physician showed a clear perception of the situation when he criticised the language of a report which claimed for a certain hospital that it was a pioneer in the investigation and treatment of disease. Treatment, he wisely said, should be given the first place. What has to be reckoned with is the aversion of many benevolent people from seeing any part of their gifts applied to purposes of education, and they are sufficiently informed to be aware that, although direct grants to the school may be forbidden, many expenses are incurred in hospitals for purposes wholly or in part clinical.

The chronic poverty of not a few hospitals is difficult to explain except upon the hypothesis that the institutions fail to secure general and whole-hearted sympathy. Why? Their domestic arrangements are the admiration of the visitor, and the almost luxurious appointments of the wards, with the kindly attitude of those who labour in them, well reflect the solicitude and respect now commonly displayed for sufferers and suffering. Concern for bodily ills is not wanting, and few men or women would hesitate long over an answer to the bald question whether our first duty is to minister to the sick at home or to send messages of religion abroad. If in the one case we have the Divine precept, in the other we are vouchsafed the Divine example. Nevertheless, missionary societies prosper while hospitals languish, and one explanation is that the aims of all concerned with the societies are identical. In the case of hospitals, belief is widespread that philanthropic and medical aims are not always in unison, and although the suggestion that patients are systematically utilised to their disadvantage is so monstrous an exaggeration as to be almost an untruth, there is sufficient interference with their privacy and comfort, and a sufficient disposition to choose them for clinical reasons to excuse the suspicion. The admission and long retention in undue numbers of patients for teaching and investigatory purposes materially curtails the accommodation for patients whose only claim is their necessity. The practice helps to bring about the deadlock which prevails commonly at many hospitals, and leads to avoidable suffering among patients kept waiting for beds.

The proposition that the professional connection with medical charity may have other than a purely benevolent origin is received with impatience. In hospital medical circles it is keenly resented. A sense of obligation to the profession inculcated among all classes by tradition, and in many individuals by gratitude, is shared largely by laymen concerned in hospital management, and none render tribute more promptly to the splendid professional work performed in the wards than those who may be described as eye-witnesses. Not the less, some of them may doubt whether the present conditions are the best for hospitals, patients, or doctors.

* Previous articles: February 20, March 6 and 13.

